# Susceptibility of Health Care Students to Measles, Paris, France

**DOI:** 10.3201/eid1709.110141

**Published:** 2011-09

**Authors:** Pierre Loulergue, Jean-Paul Guthmann, Laure Fonteneau, Jean-Baptiste Armengaud, Daniel Levy-Brühl, Odile Launay

**Affiliations:** Author affiliations: Assistance Publique Hôpitaux de Paris, Paris, France (P. Loulergue, J.-B. Armengaud, O. Launay);; Institut de Veille Sanitaire, Saint-Maurice, France (J.-P. Guthmann, L. Fonteneau, D. Levy-Brühl)

**Keywords:** health care students, measles, virus, vaccination, Paris, France, letter

**To the Editor:** A measles epidemic is currently occurring in several countries in Europe (*1*,*2*). Although most cases concern unvaccinated children and young adults, health care professionals (HCPs) are also affected. Cases occur mostly in unvaccinated persons, but also in those who have received a single dose of vaccine.

In France, the measles vaccine was introduced in the childhood-immunization schedule in 1983. Current guidelines recommend 2 doses: one at 12 months of age and the second between 13 and 24 months of age. For persons born after 1992, one catch-up dose is recommended (*3*). Coverage by >1 dose, by the age of 2 years, remained at 83%–87% during 1997–2005. The latest figures show a slight increase to 90% in 2007 (*4*).

The risk for measles in HCPs has been estimated as 13× higher than that for the general population (*5*) and is also higher among students (*6*). Vaccination against measles is recommended, not mandatory, for HCPs and health care students (HCSs) (medicine, nursing, and midwifery) who have no history of measles. The objective is to prevent transmission to a nonimmunized patient or another HCP, and from patients to susceptible HCPs. HCSs are in close and repeated contact with patients and therefore targeted by the recommendations. We conducted a cross-sectional survey in the university hospitals in Paris, France, to assess measles vaccination coverage in HCSs.

The sampling frame included 15 hospitals with an obstetrics department. All midwifery students were selected. Other students were selected through a multistage random sampling. Sampling units were selected at each stage by simple random sampling. We selected 10 hospitals at the first stage, 10 clinical wards by hospital at the second stage, and all nursing students and half the medical students by ward at the third stage. A total of 116 students were required from each profession to estimate 50% coverage with 10% precision.

Students gave oral informed consent. Information was collected by face-to-face interview. Vaccination-status was assessed from a document when available. Measles vaccination coverage was defined as the number of students with no history of measles who had received >1 dose of vaccine divided by the total number of students with no history of measles. The study was approved by the French Ethics Board and conducted from March 2009 through July 2009.

Of the 106 selected wards, 10 could not be included (clearance from the head of department was not given). Of the 488 selected students, 432 were enrolled in the study (participation rate 88.5%); 178 (41%) were medical students, 147 (34%) nursing students, and 107 (25%) midwifery students. A document confirming the student's vaccination status was available for 376 (87%) students; 38 (10.1%) had a history of measles (removed from analysis). Median age was 22 years (interquartile range 21–24 years); 74% were female. Measles vaccination was cited by 61.5% (95% confidence interval [CI] 50.0%–71.9%) as a recommended vaccination. Measles vaccination coverage was 79.3% (95% CI 71.0%–75.8%) for >1 dose and 49.6% (95% CI 40.3%–59.1%) for 2 doses (Table). When considering only the students’ accounts (without written confirmation), 1- and 2-dose vaccination coverage was 93.3% (95% CI 88.0%–96.3%) and 83.6% (95% CI 68.0%–92.4%), respectively. In multivariate analysis, younger students (<22 years of age) were more likely to have had 1 dose than older students (p<0.001).

**Table:** Age, gender ratio, and rates of measles vaccination coverage for health care students, Paris, France*

Characteristic	Medical students, n = 178	Nursing students, n = 147	Midwifery students, n = 107	Total, n = 432
Median age, y	23	22	22	22
Gender ratio, M:F	0.68	0.09	0.05	0.26
One dose, % (95% CI)	79.9 (67.1–88.6)	85.7 (67.1–88.6)	76.8 (63.1–86.5)	79.3 (71.0–85.8)
Two doses, % (95% CI)	46.3 (31.2–62.2)	66.9 (55.2–76.8)	55.7 (41.1–69.4)	49.6 (40.3–59.1)

*CI, confidence interval.

In the context of measles epidemics affecting France, and considering that the World Health Organization recommends 95% coverage of the population with 2 doses of a measles vaccine, our study shows insufficient coverage among students currently being trained as HCPs in university hospitals within the Paris area. Thus, all unvaccinated students (20.7%) and ≈5% of the 50% who have received 1 dose could be susceptible to measles. Moreover, a rather low proportion of students knew that measles vaccination was recommended, as described for HCPs (*7*), which may explain the insufficient coverage.

In France, this situation has resulted in several measles outbreaks within hospitals in recent years (*8*). Particular efforts should be made in certain units, such as pediatrics, because an early waning of maternal antibodies has been demonstrated (*9*). Measures should be taken to reinforce the vaccination policy in this well-defined group; information can be obtained and follow-up vaccinations can be provided easily during their training period. A mandatory health check could contribute to increasing the vaccination coverage (*10*).

Our study provides original data for measles vaccination coverage in HCSs in France. A similar conclusion applies to HCS-recommended vaccines for which we found insufficient coverage (<50% had received a pertussis booster in the past 15 years and only 6/27 without a history of varicella have been vaccinated against varicella). Such evaluations should be performed regularly. Although our data were collected from a representative sample of students in Paris, it is likely that the situation is qualitatively similar in other regions and therefore could contribute to future nosocomial epidemics. Mandatory vaccination of HCPs against vaccine-preventable diseases protects not only the HCP and his/her family, but also protects the patient. Increased vaccination of this group should contribute to a better control of measles outbreaks in France.
